# One-Dimensional Zinc Oxide Decorated Cobalt Oxide Nanospheres for Enhanced Gas-Sensing Properties

**DOI:** 10.3389/fchem.2018.00628

**Published:** 2018-12-17

**Authors:** Hang Zhou, Keng Xu, Yong Yang, Ting Yu, Cailei Yuan, Wenyan Wei, Yue Sun, Wenhui Lu

**Affiliations:** Jiangxi Key Laboratory of Nanomaterials and Sensors Jiangxi Normal University, Nanchang, China

**Keywords:** gas sensing, transducer function, receptor function, ethanol detection, single-crystalline

## Abstract

In this study, one-dimensional (1D) zinc oxide was loaded on the surface of cobalt oxide microspheres, which were assembled by single-crystalline porous nanosheets, via a simple heteroepitaxial growth process. This elaborate structure possessed an excellent transducer function from the single-crystalline feature of Co_3_O_4_ nanosheets and the receptor function from the zinc oxide nanorods. The structure of the as-prepared hybrid was confirmed via a Scanning Electron Microscope (SEM), X-ray diffraction (XRD), and a Transmission Electron Microscope (TEM). Gas-sensing tests showed that the gas-sensing properties of the as-designed hybrid were largely improved. The response was about 161 (R_a_/R_g_) to 100 ppm ethanol, which is 110 and 10 times higher than that of Co_3_O_4_ (R_g_/R_a_ = 1.47) and ZnO (R_a_/R_g_ = 15), respectively. And the as-designed ZnO/Co_3_O_4_ hybrid also showed a high selectivity to ethanol. The superior gas-sensing properties were mainly attributed to the as-designed nanostructures that contained a super transducer function and a super receptor function. The design strategy for gas-sensing materials in this work shed a new light on the exploration of high-performance gas sensors.

## Introduction

Metal oxides, as a type of predominant gas sensing material, have provoked considerable attention because of their low cost, excellent electrical properties and controllable preparation (Korotcenkov, 2007; Li et al., 2016; Dong et al., 2018). It is well accepted that the gas-sensing processes of metal oxides, contain not only gas diffusion and gas reaction on its surface, but also signal transformation that transforms the surficial chemical signal into an electrical resistance variation (Rai et al., 2013). In this regard, gas-sensing performances of metal oxides depend largely on the structure factors of sensing materials such as surface area, morphology, as well as signal transmission channels (Zhu et al., 2018). Nowadays, many studies have reported that the gas-sensing properties of metal oxides can be improved by the creation of heterojunctions, which can provide tunable morphologies and compositions (Miller et al., 2014; Cao et al., 2017; Gong et al., 2018). For example, the In_2_O_3_/Co_3_O_4_ composites synthesized by Mirzaei et al., exhibited superior gas-sensing performances than pristine In_2_O_3_ did (Mirzaei et al., 2017). However, up to now, most related studies have been based on mechanical mixed composites or inhomogeneous structures, resulting in randomly distributed heterojunctions. In particular, the gas-sensing processes of composites that contain gas diffusion, gas reaction, and signal transformation still need to be further improved.

In this paper, ZnO nanorods anchored on the surface of Co_3_O_4_ nanospheres were synthesized by an epitaxial growth method. ZnO and Co_3_O_4_ have been widely investigated in the field of gas-sensing application, because of their chemical and physical stability and abundance in raw materials (Tan et al., 2017; Zhu and Zeng, 2017). We chose the ZnO nanorods and Co_3_O_4_ microspheres because of their large surface area for gas reaction and because of the effective accesses for gas diffusion (Park et al., 2016). Moreover, the Co_3_O_4_ nanospheres consisted of single-crystalline porous nanosheets which provided effective electrical pathways for charge-carrier transfer and was more beneficial to the signal transformation process (Singh et al., 2011; Zhang et al., 2015). Therefore, these ZnO nanorods acted as “trigger hairs,” while Co_3_O_4_ microspheres acted as “channels.” And the p-n heterojunctions between ZnO and Co_3_O_4_ also led to an extended depletion region and a high initial resistance, which is more beneficial for the transducer function (Li et al., 2015).

## Experiment

### Synthesis of ZnO/Co_3_O_4_ Hybrid

All the chemical reagents were purchased from Sinopharm Chemical Reagent and used without further purification. During the synthesis processes (Xu et al., 2017), a mixed solution (80 ml) composed of distilled water and ethylene glycol (1: 79) was added at 0.05 g PVP, as well as 1 g cobalt acetate. The mixed solution was then stirred and poured into a Teflon-lined stainless-steel autoclave (100 mL). The resulting precipitate was collected and washed repeatedly at least seven times and thereafter maintained at 180°C for 12 h. The final powder was then annealed at 350°C for 2 h in air atmosphere. To prepare the ZnO/Co_3_O_4_ hybrid, 0.03g as-prepared Co_3_O_4_ and 0.32 g zinc acetate dehydrate was added in methanol through the assistance of ultrasonication. A KOH methanol solution (60 ml) was then also added to the above mixture. After 2 h of stirring, the mixture was centrifuged at 3,000 rpm and washed several times. The powder obtained, was then was added into a mixed solution composed of Zn(NO_3_)_2_ (0.89 g) and HMT (0.42 mol/L) by vigorous stirring. The final as-designed hybrid was obtained after being refluxed at 95 °C for 6 h.

### Sensor Fabrication and Measurements

To fabricate a gas sensor, a certain number of as-prepared samples were uniformly dispersed in ethanol, by ultrasonication for 15 min, to form a suspension. The suspension was then coated onto a ceramic flat, which had been coated with Pt interdigitated electrodes and a Pt heat element (about 500 μm). The obtained film was sintered at 500°C for 2 h and aged at 300°C for 3 days in air atmosphere. Gas-sensing performances were measured in a static testing instrument which was purchased from Wuhan Hua Chuang Rui Ke Co. Ltd. This equipment includes a test chamber (about 30 L in volume) and a personal computer. During the gas-sensing tests, the target gases were introduced into the test chamber by injecting their corresponding liquid, with a calculated amount, which was then vaporized by a heater. The desired concentrations of the testing gases are calculated by the following formula:

$$\begin{array}{l} Q=\frac{V\times \varphi \times M}{22.4\times d\times \rho }\times 10^{-9}\times \frac{273+T_{R}}{273+T_{B}} \end{array}$$

where Q (ml) is the liquid volume of the volatile compound, V (ml) is the volume of the testing chamber, ϕ is the required gas volume fraction, M (g. mol^−1^) is the molecular weight, d (g. cm^−3^) is the specific gravity, and ρ is the purity of the volatile testing liquid, T_R_ and T_B_ (°C) are the temperatures at ambient and the test chamber, respectively. For example, the liquid volume of ethanol is calculated at about 13.05 μL that corresponds to 100 ppm. Two electric fans installed in the chamber were used to make the test gas homogeneous. After a few minutes, the chamber was lifted to introduce ambient air (humidity: 16~22%). In the meantime, the resistances of the sensors were recorded by a personal computer.

## Results and Discussion

**Figure 1:**
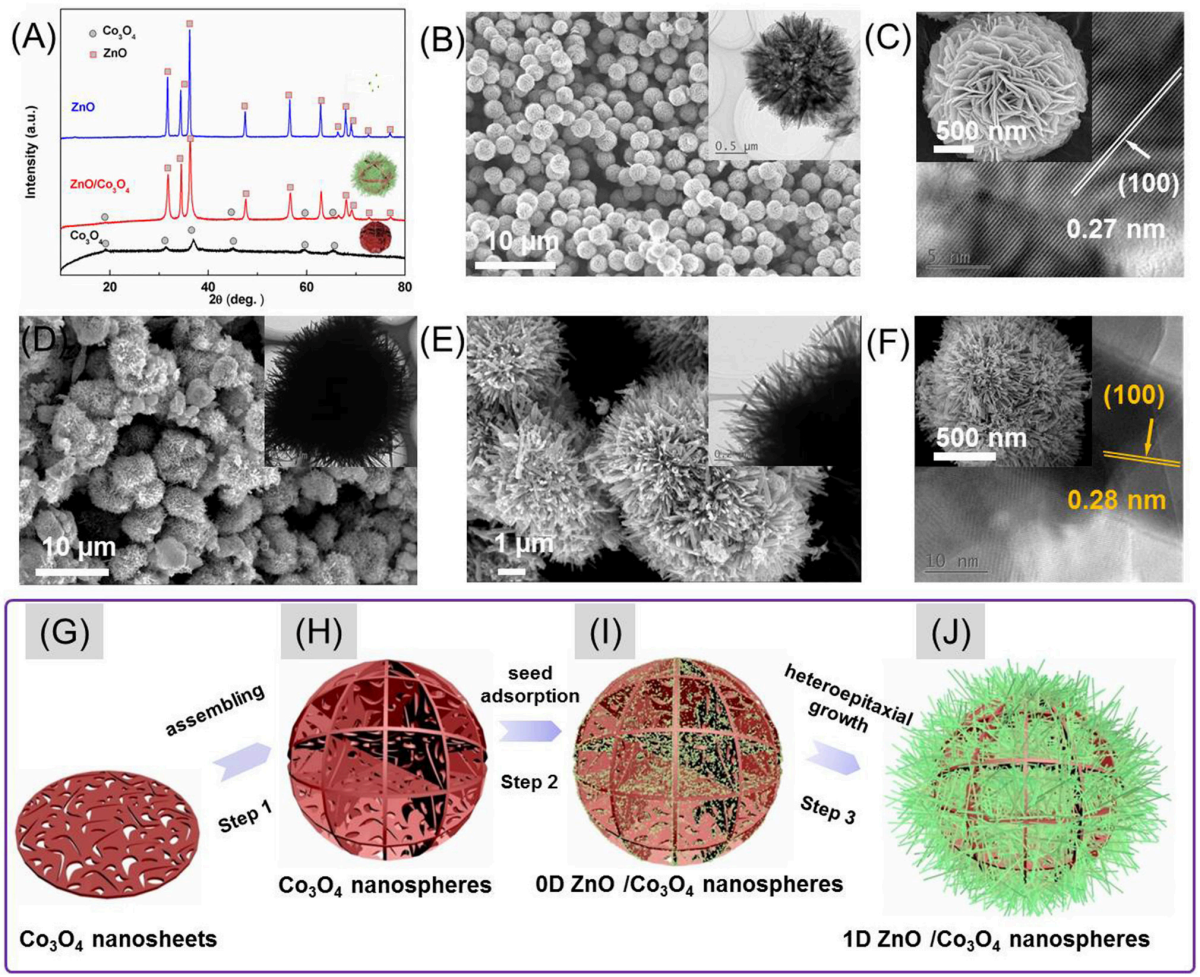
**(A)** XRD spectra of Co_3_O_4_, ZnO, and ZnO/Co_3_O_4_. **(B,C)** SEM and TEM (inset) of Co_3_O_4_. **(D–F)** SEM and TEM (inset) of ZnO/Co_3_O_4_. **(G–J)** Schematic illustration of the formation processes of ZnO/Co_3_O_4_ hybrid.

The crystalline phases of as-prepared samples were characterized by XRD (Figure 1A). As can be seen, beside the peaks that belong to Co_3_O_4_ (JCPDS No. 42-1467), all the diffraction peaks left in the pattern of hybrids can be indexed to the reflections of ZnO (JCPDS No. 36–1451).The morphology of Co_3_O_4_ is shown in the Figures 1B,C. It is seen that the Co_3_O_4_ is composed of numerous uniform microspheres with a diameter of 2–4 μm. These microspheres are composed of many crossed 2D nanosheets with numerous pores, as shown in the enlarged images (Figure 1C). It is worth mentioning that the individual nanosheets containing numerous pores are single crystals, since each nanosheet consisted of coherent lattice fringes regardless of the pore in the inset of Figure 1C. The single crystal can provide effective electrical pathways for charges, which benefits the gas-sensing performances (Meng et al., 2015). Accordingly, the morphology of ZnO/Co_3_O_4_ was also characterized (Figures 1D–F). Figure 1D reveals that the ZnO/Co_3_O_4_ hybrids were also composed of numerous microspheres. However, its diameter was much larger than that of pristine Co_3_O_4_. The enlarged images (Figures 1E,F) reveal a hedgehog-like structure of ZnO/Co_3_O_4_ where ZnO nanorods were formed on the surface of Co_3_O_4_ as trigger hairs. The ZnO/Co_3_O_4_ hybrids were further characterized through EDS (Figure S1). Only O, Zn, Co, together with C elements were detected. The average atomic ratio of Zn and Co was about 1:6.5. The distributions of O, Zn and Co are presented in Figures S1A–D. It was revealed that the elements O, Zn, and Co were distributed homogeneously. The growth processes of ZnO on the surface of cobalt oxide were displayed under different time conditions as shown in Figure S2. The preparation processes of ZnO/Co_3_O_4_ are thus illustrated in Figures 1G,H. As seen in step 1, the as-obtained Co_3_O_4_ microspheres are composed of many single-crystalline porous nanosheets. These Co_3_O_4_ microspheres were then anchored with ZnO crystal seeds by soaking them into a pre-prepared ZnO colloid solution as shown in step 2 (Figure 1I). Finally, via a heteroepitaxial growth process, ZnO nanorods were grown on the surface of Co_3_O_4_ microspheres (Figure 1J).

**Figure 2:**
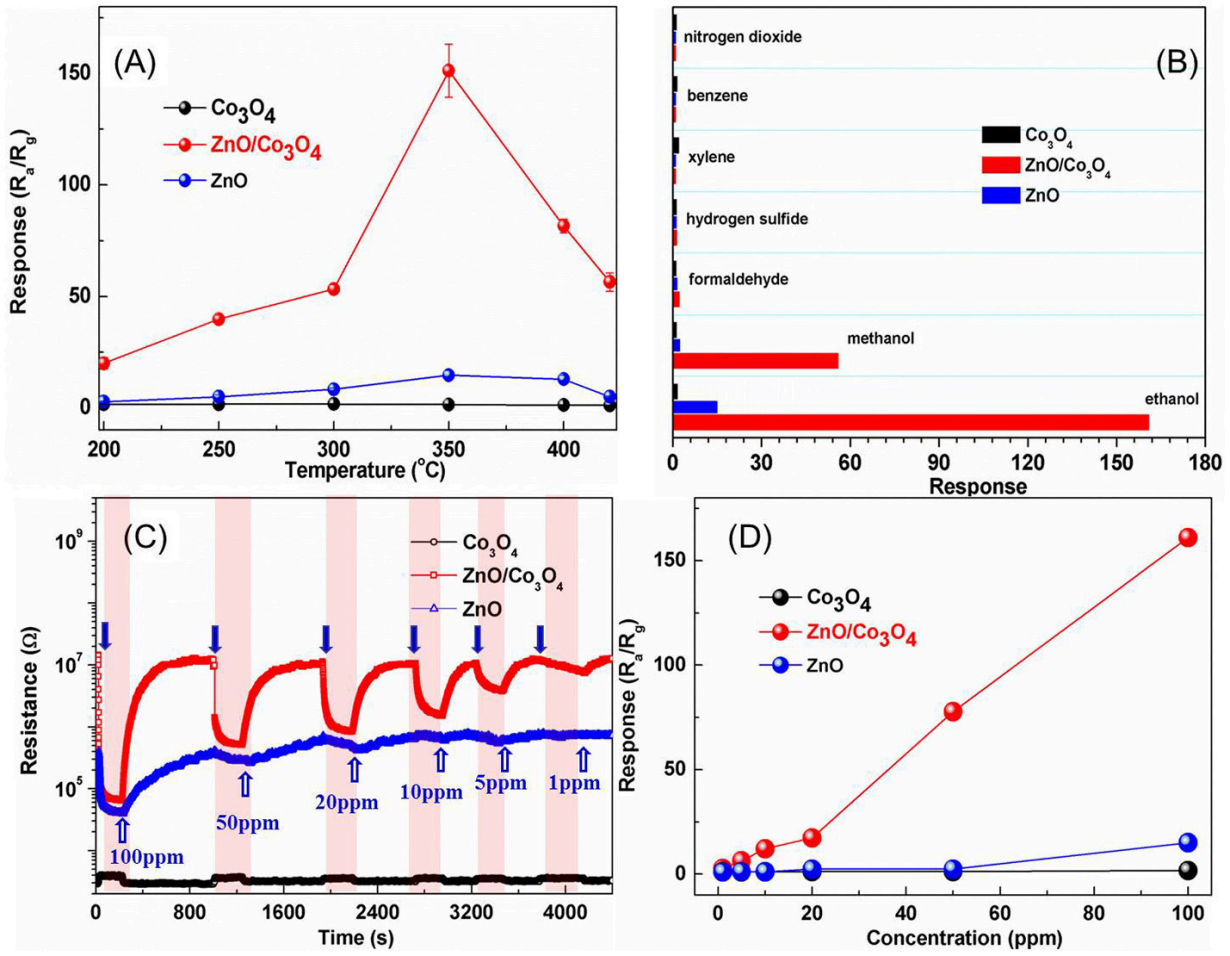
**(A)** Responses of Co_3_O_4_, ZnO, and ZnO/Co_3_O_4_ microspheres to 100 ppm ethanol at different operating temperatures. **(B)** Responses to different gases with a concentration of 100 ppm. **(C)** The dynamic gas response patterns to ethanol with different concentration. **(D)** The response as a function of concentration.

The responses of these sensors to 100 ppm ethanol at different operating temperatures are shown in Figure 2A. It can be observed that the optimal operating temperature is 300°C for ZnO/Co_3_O_4_. The response was about 161 (R_a_/R_g_) to 100 ppm ethanol, which is 110 and 10 times higher than that of Co_3_O_4_ (R_g_/R_a_ = 1.47) and ZnO (R_a_/R_g_ = 15), respectively. Gas-sensing tests of ZnO/Co_3_O_4_ toward other gases (100 ppm) were carried out as shown in Figure 2B. It was found that the responses to ethanol was much higher than to other gases. This could be due to the different volatilities and chemical properties of gases, which prompt the sensors to exhibit different adsorption and catalytic performances toward them. Therefore, the polarity, molecular weight and structure of these gases can exert great effect on the gas-sensing response. The difference between ethanol and methanol in this work may be the molecular weight. Alcohol with larger molecules can be more easily adsorbed and can release more electrons. On the other hand, different constituents and structures with various surface properties are also believed to influence the selectivity of sensing material, due to the diverse chemisorption abilities, which induce different selectivity behaviors between Co_3_O_4_, ZnO/Co_3_O_4_, and ZnO. The responses to 1~100 ppm ethanol were tested as shown in Figure 2C. Evidently, exposure to ethanol gas led to an increase in resistance for Co_3_O_4_, while it led to a decrease for ZnO and ZnO/Co_3_O_4_, indicating that Co_3_O_4_ exhibits a p-type semiconducting behavior while ZnO and ZnO/Co_3_O_4_ exhibits an n-type behavior. Figure 2D present the responses of Co_3_O_4_ and ZnO/Co_3_O_4_ as a function of the ethanol concentrations (1–100 ppm), from which the gas responses increase almost linearly with the gas concentration.

The real-time responses of Co_3_O_4_, ZnO, and ZnO/Co_3_O_4_ to 100 ppm ethanol with three cycles are displayed in Figure S3. The resistance patterns reveal the similar continuous recycles, indicating all of the samples exhibit good repeatability. The response and recovery time are also important parameters in the fields of gas detection. It was revealed that the response time of Co_3_O_4_, ZnO, and ZnO/Co_3_O_4_ are 6, 17, and 13 s, respectively. And the recovery times of Co_3_O_4_, ZnO, and ZnO/Co_3_O_4_ were 12, 405, and 168 s, respectively. The fast response and recovery of Co_3_O_4_ can be attributed to its single-crystalline porous structure which benefits the gas-diffusion process and charge-transfer process. Compared with pure ZnO, the response and recovery process of ZnO/Co_3_O_4_ were accelerated because of its super single-crystalline porous structure. In addition, the response and recovery speeds of ZnO/Co_3_O_4_ to ethanol with low concentrations was much faster than those in ethanol with high concentrations, because of the slow diffusion speed of ethanol molecules to the active site at a low concentration.

The excellent gas-sensing properties of the ZnO/Co_3_O_4_ to ethanol, are mainly due to the many benefits of the as-designed structure. First, the ZnO nanorods and Co_3_O_4_ microspheres provide numerous channels for gas diffusion and an extremely high surface area for gas reaction. Furthermore, the single-crystalline feature of Co_3_O_4_ nanosheets provides a fast transport of charge carrier, another main factor to improve the signal transformation process of a hybrid. Moreover, the p-n heterojunctions between ZnO and Co_3_O_4_ can enlarge the response signal. Because the conduction band edge of Co_3_O_4_ is more negative than that of ZnO, band bending, and depletion regions are formed at the heterojunctions (Jana et al., 2015). By increasing the initial resistance, due to the as-established depletion region, the modulation of resistance will become more evident (Kim et al., 2017).

## Conclusions

In this paper, ZnO nanorods anchored on the surface of Co_3_O_4_ nanospheres were synthesized. ZnO nanorods and porous Co_3_O_4_ microspheres provided numerous channels for gas diffusion and a large surface area for gas reaction. The Co_3_O_4_ nanospheres consisted of single-crystalline porous nanosheets to provide effective electrical pathways for charge-carrier transfer. And the p-n heterojunctions between ZnO and Co_3_O_4_ also led to an extended depletion region and a high initial resistance. As expected, this as-designed hybrid exhibited excellent gas-sensing properties with an extremely high response (R_a_/R_g_ = 161) and a high selectivity to ethanol.

## Author Contributions

HZ performed the experiments and analyzed the data with the help from YY, TY, CY, WW, YS, and WL. KX wrote the manuscript with input from all authors. All authors read and approved the manuscript.

### Conflict of Interest Statement

The authors declare that the research was conducted in the absence of any commercial or financial relationships that could be construed as a potential conflict of interest.
