# Supplementary material for: One-Dimensional Zinc Oxide Decorated Cobalt Oxide Nanospheres for Enhanced Gas-Sensing Properties
Source: Front Chem. 2018 Dec 17;6:628. doi: 10.3389/fchem.2018.00628 (PMC6304346; doi:10.3389/fchem.2018.00628)
Supplement: Supplementary file 1 [file Data_Sheet_1.PDF]

Supplementary Material

One-Dimensional Zinc Oxide Decorated Cobalt Oxide Nanospheres for Enhanced Gas-Sensing Properties

Hang Zhou[a], Keng Xu[*a], Yong Yang[a], Ting Yu[a], Cailei Yuan[*a]

Wenyan Wei[a], Yue Sun[a], Wenhui Lu[a]

[a]Jiangxi Key Laboratory of Nanomaterials and Sensors, Jiangxi Normal University, Nanchang 330022, Jiangxi, P. R. China

Corresponding Author. Tel:0791-88120370;

Email address: xukeng@163.com; clyuan@jxnu.edu.cn

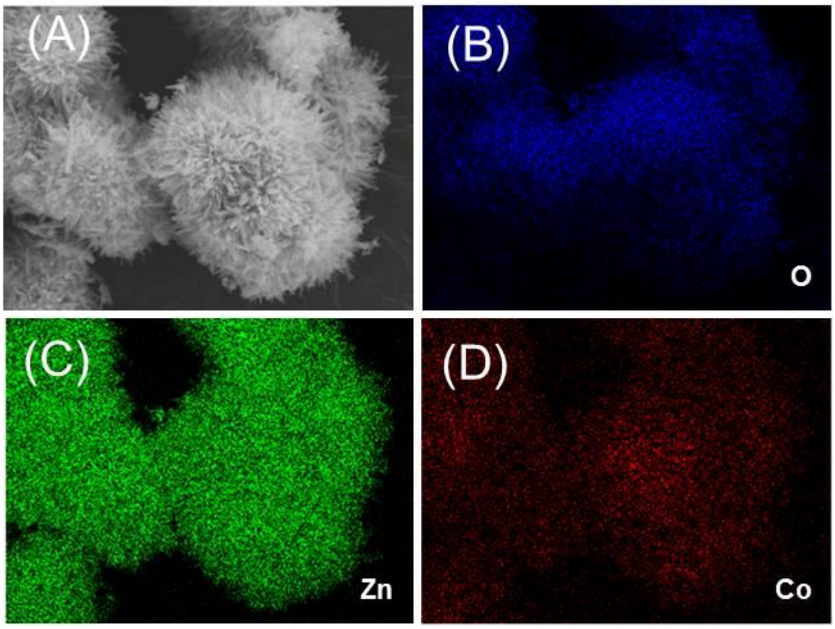

FIGURE S1 EDS elemental mapping of O, Zn, and Co.

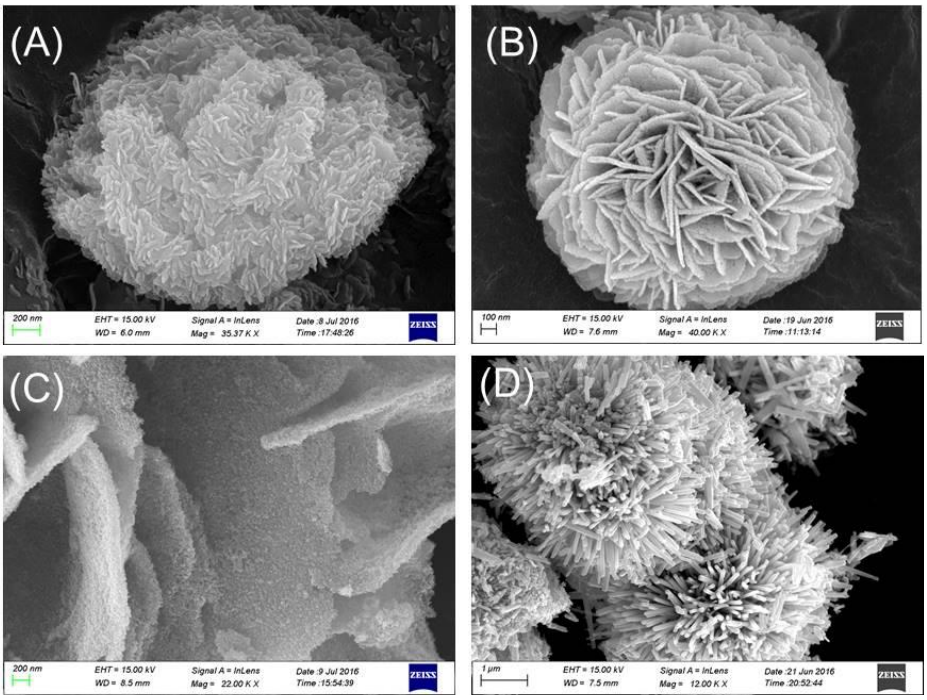

FIGURE S2 SEM images of growth processes under different time conditions during the (A) Co_3O_4 nanosheets; (B) Co_3O_4 nanosphere; (C) 0D ZnO/ Co_3O_4 nanosphere; (D) 1D ZnO/Co_3O_4 nanosphere.

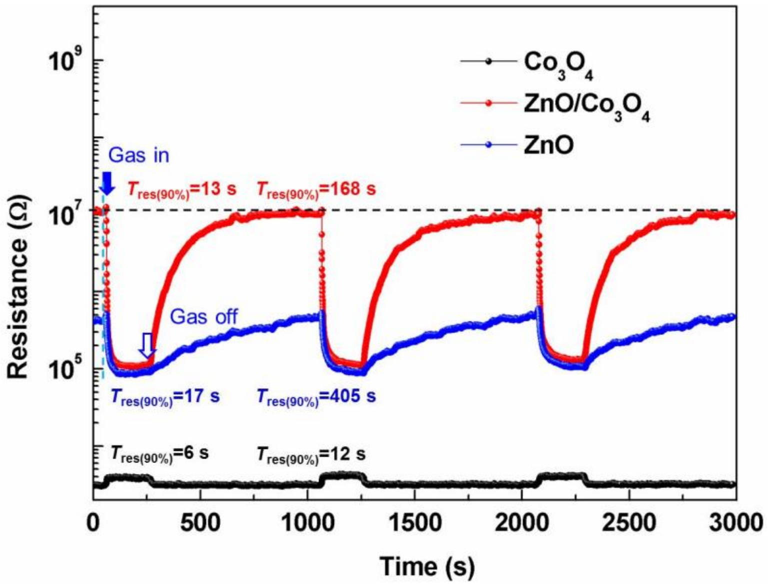

FIGURE S3 Response-recovery detail pattern of Co_3O_4, ZnO and ZnO/Co_3O_4 to 100 ppm ethanol at their optimal operating temperature with dynamic response-recovery cycles to 100 ppm ethanol.
